# Vaccines for human fungal diseases: close but still a long way to go

**DOI:** 10.1038/s41541-021-00294-8

**Published:** 2021-03-03

**Authors:** Lorena V. N. Oliveira, Ruiying Wang, Charles A. Specht, Stuart M. Levitz

**Affiliations:** Department of Medicine, University of Massachusetts Medical School, Worcester, MA USA

**Keywords:** Diseases, Immunology

## Abstract

Despite the substantial global burden of human fungal infections, there are no approved fungal vaccines to protect at risk individuals. Here, we review the progress that has been made and the challenges that lie ahead in the quest towards efficacious fungal vaccines. In mouse studies, protection has been achieved with vaccines directed against fungal pathogens, including species of *Candida, Cryptococcus*, and *Aspergillus*, that most commonly cause life-threatening human disease. Encouraging results have been obtained with vaccines composed of live-attenuated and killed fungi, crude extracts, recombinant subunit formulations, and nucleic acid vaccines. Novel adjuvants that instruct the immune system to mount the types of protective responses needed to fight mycotic infections are under development. Candidate vaccines include those that target common antigens expressed on multiple genera of fungi thereby protecting against a broad range of mycoses. Encouragingly, three vaccines have reached human clinical trials. Still, formidable obstacles must be overcome before we will have fungal vaccines licensed for human use.

## Introduction

The global burden of serious fungal diseases is increasing as a direct consequence of the burgeoning number of immunocompromised persons^1^. Risk factors for invasive fungal infections include infection with the human immunodeficiency virus (HIV), immunosuppressive therapy to prevent organ transplant rejection, biological immunomodulatory agents to treat autoimmune diseases, bone marrow suppressing cancer chemotherapies, indwelling devices such as intravenous catheters, and long-term hospitalization, especially with receipt of broad-spectrum antibiotics^1,2^. Even with the availability of antifungal drugs, the burden of life-threatening fungal infections is thought to exceed one million deaths annually, although numbers are difficult to estimate due in part to inadequate availability of diagnostic tests and disease reporting^3,4^. Superficial mycoses, such as dermatophytic infections of the skin and nails, affect more than 25% of the population worldwide, although they are generally treatable^3^. The annual medical cost of fungal diseases is estimated to exceed 7.2 billion dollars in the United States alone^5^.

The substantial morbidity and mortality rates highlight the relevance of developing effective vaccines to control fungal pathogens. Despite efforts, unfortunately, there are no licensed vaccines available to prevent and control human invasive fungal infections. In this review, we outline the advances and challenges toward the development of fungal vaccines, providing examples of potential targets and promising vaccine strategies.

### Challenges and efforts toward developing fungal vaccines

Vaccines are considered one of the greatest achievements in medicine^6^. For example, their use led to eradication of smallpox, and substantially reduced poliomyelitis, measles, diphtheria, and pneumococcal infections^7^. Additionally, according to the World Health Organization (WHO), vaccines are being successfully used against more than 25 debilitating or life-threatening diseases, including tetanus, rabies, influenza, meningitis, cholera, rubella, and hepatitis B.

For protection against communicable diseases, vaccines are among the most cost-effective measures available. Vaccines for many infectious diseases, including invasive mycoses, however, are not available^6,8^. Fungal diseases often have a poor prognosis stemming in part from the limited arsenal of antifungal drugs compounded by the increasing occurrence of antifungal resistance^9^. The lack of reliable diagnostics for many fungal diseases can lead to delays in treatment^3^. Given that, worldwide efforts are being made to develop vaccines against fungal pathogens^2,8,10,11^. Still, formidable challenges remain, many of which, along with their potential solutions, are listed in Table 1.

**Table 1:** Challenges associated with fungal vaccine development.

Challenges	Potential solutions
Population most at risk is immunosuppressed	Vaccinate prior to anticipated immunosuppression.
	Augment specific immune system responses less affected by immunosuppression.
	Transfer protective lymphocytes to patient.
	Improve adjuvants.
Diverse infection sites in the host	Utilize delivery systems or adjuvants that drive the immune response at multiple sites of infections.
Intraspecies and interspecies antigenic variation among fungi	Target multiple epitopes with multivalent vaccines.
Similarities between Fungi and Animalia kingdoms	Target structures existing only in fungi, such as the cell wall.
	Use protein antigens unique to fungi to minimize autoimmune responses.
Translation from animal models to humans	Test the candidate vaccine in multiple animal models.
	Perform ex vivo studies with human cells.
Formulation	Select a delivery system and/or adjuvants to optimize protective responses and safety.
Commercialization	Attract interest and investments from governmental agencies, non-governmental organizations, and biopharmaceutical companies.

As mentioned, the immunocompromised population is at highest risk for serious fungal infections. However, immunological impairment poses challenges with respect to both the efficacy and safety of vaccines. In this regard, the high immunogenicity of live vaccines makes them perhaps most likely to elicit protective responses, but must be used with caution because of the potential risk for infections from the vaccine itself. On the other hand, inactivated whole organism and subunit vaccines are safer; however, immunocompromised individuals are less likely to respond to vaccination due to their debilitated immune status^12^. Efforts are being made to improve adjuvants and vaccine formulations to elicit stronger protective responses, and to target those immune response pathways that may not be compromised^6^. Another potential solution is vaccinating individuals prior to their anticipated immunosuppression when the immune system is still functionally effective. Examples include persons awaiting solid organ transplantation and individuals with HIV infection, who have relatively high CD4^+^ T cell counts.

An additional challenge to developing a vaccine is translating preclinical studies in animals to humans. Most in vivo vaccine studies are initially conducted in inbred mice, as they are relatively inexpensive and have a well-defined immune system. However, inherent differences in murine and human immune responses are still a concern and caution needs to be taken with regard to extrapolating efficacy data across species^13^. Even with the attempt to reproduce aspects of human disease, inbred mice lack genetic diversity. Moreover, laboratory mice are generally housed under conditions whereby they have limited exposure to environmental fungi, such as airborne spores and thus may not be exposed to fungal antigens commonly encountered in the “real world”. To minimize these drawbacks, prior to clinical studies, vaccine candidates ideally should be tested in multiple animal models.

Reactogenicity and safety must be investigated in pre-clinical models as a prerequisite to clinical trials^14^. At this point, converting a vaccine candidate into one approved for use in humans entails financing clinical trials and product manufacture. For those fungal infections that mostly affect populations living in resource-limited areas, the interest of pharmaceutical companies may be limited and vaccine commercialization could require attracting investments of governmental and non-governmental organizations^11^. That said, fungal pathogens are a major public health concern worthy of global attention, and funding incentives for preventive fungal vaccines are urgently needed.

### Fungal cell wall structure

Fungi and animals are phylogenetically grouped in the same Eukaryotic domain^15^. Then not surprisingly, many similarities exist between fungal and human cells; these become important considerations for drug discovery and treatment of fungal diseases. A major difference between the Fungi and Animalia kingdoms is the presence of the cell wall on almost all fungal cells^16^. Consequently, the proteins that synthesize and remodel the cell wall are important drug targets. Moreover, fungal cell wall components are recognized by the innate immune system in humans^17,18^, leading to adaptive and trained immune responses. As discussed below, an area of research is exploiting this response in vaccine development.

**Fig. 1:**
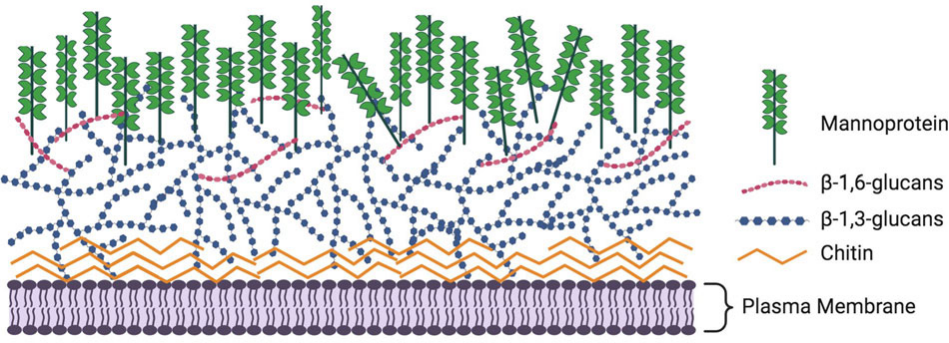
Schematic model of the fungal cell wall. This model shows the three basic components of the cell wall present in almost all fungal pathogens. Mannoproteins have polymers of mannose that decorate the proteins through O-linkages or N-linkages and are predominately found in the outer portion of cell wall. β-glucans are the most abundant constituent of the cell wall and tend to be in the middle with β-1,3-glucans forming the scaffold and β-1,6-glucans forming the branches, while chitin is found in the inner portion of the cell wall and linked to β-1,3-glucan. Other components of the cell wall in some fungi include α-glucan, galactomannan, chitosan, and melanin. In addition, the cryptococcal capsule is linked to the cell wall. Figure created with BioRender.com.

The cell wall is composed mainly of conserved crosslinked carbohydrate polymers and mannoproteins that are recognized by pattern recognition receptors on immune cells of the host, notably monocytes, macrophages, and dendritic cells^19,20^. The most abundant components of the fungal cell wall are mannoproteins and β-glucans, followed by chitin/chitosan. Mannoproteins are predominately found in the outer portion of cell wall, β-glucans tends to be in the middle, while chitin/chitosan trends towards the inner portion of the cell wall. These constituents of the cell wall are found in practically all invasive fungal pathogens (reviewed in the refs. ^18,21^). An overview of the fungal cell wall components is provided in Table 2 and Fig. 1.

**Table 2:** Major components of fungal cell walls.

Fungal cell wall components	Structure composition	Location
Chitin	Homopolymer of N-Acetylglucosamine that provides structural stability	Inner portion of cell wall, adjacent to the plasma membrane
Chitosan	Deacetylated chitin	Inner portion of cell wall, intermingled with chitin
β-glucan	Homopolymers of glucose with β-1,3-glucans forming the scaffold and β-1,6-glucans forming the branches	Middle portion of cell wall, between chitin and mannans
Mannoprotein	Mannose chains of varying lengths and configurations added to fungal proteins via N-linkages or O-linkages	From anchorage in plasma membrane to outer portion of cell wall

Some fungal cell walls also contain galactomannan, α-glucan and melanin^21^. In addition, it is important to highlight the presence of a polysaccharide capsule on *Cryptococcus* spp., composed primarily of glucuronoxylomannan (GXM) and galactoxylomannan; the capsule is a dominant virulence factor^22^. Structurally, the capsule envelops the cell wall and, among its several roles, “masks” recognition of cell wall ligands by pattern recognition receptors thus interfering with the development of immunity^16^. Finally, fungi release extracellular vesicles containing proteins, lipids, and nucleic acids. In addition to secretion into the extracellular milieu, extracellular vesicles may traffic to the cell wall and contribute to cell wall remodeling and enable surface expression of proteins^23^.

Compounding the challenges encountered by interspecies differences in the fungal cell wall structure, the host faces dynamic changes in the distribution and amount of fungal cell wall components that accompany morphological changes during infection. External stress events, transition between yeast and hyphal growth, and the process of cellular division can impact the cell wall to decrease host recognition, impair inflammatory responses, and increase fungal virulence^16,24^.

### Fungal vaccine categories and adjuvants

Fungal vaccines can be divided into several broad categories based upon their composition, ranging from multiple to single antigens: whole organism vaccines (live-attenuated or killed fungal cells), crude extracts (fractions derived from cells and medium of fungal cultures), purified subunit vaccines (proteins, peptides), and nucleic acids (RNA and DNA) encoding the antigen(s) of interest (Table 3). Approaches using dendritic cells pulsed ex vivo with antigen(s) are not practical for routine immunizations, but have potential for use in therapeutic vaccines for patients with refractory diseases^25^. As discussed above, whole organism vaccines generally have strong immunogenicity but often at the cost of increased side effects, including risk of infection when live-attenuated vaccines are given to immunocompromised populations. Thus, many investigators have focused on identifying antigens which, either in native or recombinant form, can be used in subunit fungal vaccines.

**Table 3:** Overview of the advantages and disadvantages of the major fungal vaccine categories.

Vaccine Category	Advantages	Disadvantages	Representative vaccines
Live-attenuated	Strong and long-lasting immunogenicity; manufacturing processes are straightforward	Risk of sustained infection in immunocompromised population; reactogenicity; autoimmunity	Mutant *C. neoformans* strain lacking the enzyme sterylglucosidase 1^59^
Killed fungus	Cannot cause infection; stabler than live-attenuated vaccines; manufacturing processes are straightforward	Elicit less strong immune responses compared to live vaccines; reactogenicity; autoimmunity	Formalin-killed spherule vaccine for coccidioidomycosis^75^
Fungal extracts	Contain numerous multivalent antigens	Reactogenicity; autoimmunity	Glucan particles containing *Cryptococcus* alkaline extracts^53^
Purified proteins, peptides, carbohydrates, and lipids	Fewer antigens minimizes the potential side effects	Narrow immune response due to fewer antigens; adjuvants are needed; careful epitope selection, antigen design and purification are required	NDV-3A vaccine (containing the recombinant N-terminus of *C. albicans* agglutinin-like sequence 3 protein)^44^
Nucleic acid-encoded delivery of antigen(s): DNA or RNA vaccine	Fast manufacturing process; strong immune responses	Risks of eliciting unintended immune reactions; strict temperature requirements for storage	DNA vaccine encoding cell wall antigen Mp1p against *Penicillium marneffei* infection^81^

Subunit vaccines require adjuvants, compounds which enhance antigen immunogenicity by promoting adaptive immune responses. Most vaccines in clinical use mediate antibody-dependent protection via mechanisms that include opsonophagocytosis and neutralization of viruses and toxins. Traditional adjuvants which promote antibody responses, particularly alum, have been effective for these purposes. However, because CD4^+^ T helper cell (Th)-mediated immunity is paramount for defending against many mycoses, adjuvants which elicit strong Th1 or Th17 cell-mediated responses have been proposed for inclusion in fungal vaccines^26^. Some adjuvants used in experimental models to stimulate Th responses, such as complete Freunds adjuvant, are too toxic for routine use in humans but may be useful in experimental vaccine studies to demonstrate proof of principle^27^. Other adjuvants, such as CpG oligodeoxynucleotides, may be more translationally relevant as they have been used in human vaccines^6^.

We and others have proposed exploiting components of fungal cell walls, such as β-1,3-glucans and mannans, as adjuvants, reasoning that the immune response generated will mimic the type of protective immune response seen in natural infection^18^. One such approach has been to package fungal antigens into glucan particles (GPs). GPs are hollow, porous yeast cell wall shells manufactured from *Saccharomyces cerevisiae*, and primarily composed of β-1,3-glucan^28^. GPs are recognized by Dectin-1 and are also potent activators of the complement pathway^29^. Mice immunized with GPs loaded with the model antigen ovalbumin developed long-lasting antigen-specific antibody and Th1-biased and Th17-biased CD4^+^ T cell responses^28^. Mice vaccinated with GPs formulated with fungal antigens were protected following challenges with a range of fungal pathogens^30–33^. Using a related approach, protective vaccines consisting of *Coccidioides* and *Blastomyces* antigens packaged into glucan-chitin particles (GCP) have been described^34^. GCPs are similar to GPs except they are produced from the yeast *Rhodotorula mucilaginosa* and contain extra chitin in their cell walls.

### Potential fungal vaccines

**Fig. 2:**
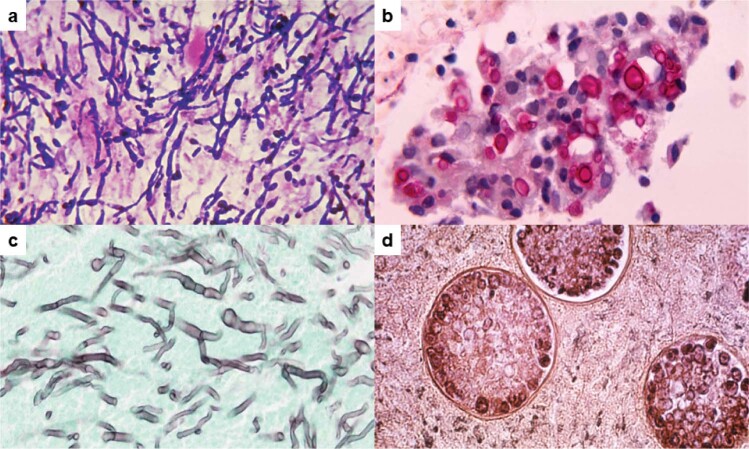
Examples of the diversity in fungal morphology in human tissue from patients with mycoses. **a** Tissue Gram stain of *C. albicans* from a patient with endocarditis. Hyphae (elongated cells), pseudo-hyphae (sausage-shaped cells) and yeasts (oval cells, some with buds) stain deep purple. *Candida* cells average 2–8 microns in diameter. **b** Mucicarmine stain of *C. neoformans* in the lungs of a patient with pulmonary cryptococcosis. Budding yeast cells with capsules that stain rose red are present. Yeast cells average about 5 microns in diameter without capsule. Capsular thickness is variable, typically ranging from 1 to 10 microns. **c** Grocott’s methenamine silver stain of *A. fumigatus* from a patient with invasive pulmonary aspergillosis. Septate hyphae with “Y”-shaped branching that stain silvery black are present. Average hyphal diameter is about 3 microns. **d** Periodic acid-Schiff stain of *C. immitis* from a patient with coccidioidomycosis. Three spherules, each containing endospores, are present. Spherules and endospores range in diameter from 10 to 100 microns and 2 to 5 microns, respectively. A single spherule can contain hundreds of endospores. Photo image credits. **a**, **b**, and **d**: Centers for Disease Control Public Health Image Library. **c:** Wikimedia Commons.

*Candida, Cryptococcus, Aspergillus*, and *Pneumocystis* are the most common fungal genera causing invasive human infections. Endemic dimorphic fungi, such as *Histoplasma*, *Coccidioides*, *Paracoccidioides*, and *Blastomyces* also cause invasive mycosis^1–3^. Examples of the disparate morphologies of fungi in human tissue are shown in Fig. 2. Other fungi that cause serious infections include species of *Mucor*, *Sporothrix*, *Scedosporium*, and *Fusarium*^1^. However, for these and other relatively rare fungi, clinical testing of pathogen-specific vaccines presents obvious logistical difficulties. In this section, we review vaccine candidates against the fungal genera responsible for most cases of invasive mycoses.

### *Candida* sp.

*Candida* species commonly colonize humans as commensal organisms; however, in situations of immunosuppression they can opportunistically become pathogens. *Candida albicans* and non-*albicans* species are the most common cause of life-threatening invasive fungal infections^9,35^. Globally, an estimated 700,000 persons a year suffer from invasive candidiasis^4^, with an associated mortality that may exceed 50%^3^. Furthermore, *Candida* can also cause mucocutaneous infections, such as vulvovaginal candidiasis which, while rarely lethal, are associated with significant morbidity^9^. For example, vulvovaginal candidiasis can have a profoundly negative impact on quality-of-life. It is estimated that the majority of women experience vulvovaginal candidiasis at least once in their lifetime and many women suffer from recurrent disease^36^. Importantly, the emergence of drug resistant strains, such as *C. auris*, is a public health concern. Thus, the need for new therapies and protective vaccines against *Candida* has increased.

With mouse models of vulvovaginal and/or systemic candidiasis, the efficacy of anti-*Candida* vaccines has been demonstrated targeting virulence factors, and *Candida* virulent forms such as hyphae and cell wall antigens, with a variety of formulations, including live attenuated (generally strains with impaired yeast-hyphae conversion^37^); recombinant proteins (using surface-located or adhesion proteins^38,39^); cell wall extracts, extracellular vesicles^40^, and glycoconjugates^41^. However, morphological, phenotypic, and genetic variability among *Candida* species poses a challenge to vaccine development. In addition, as *Candida* is a common human commensal, there is a theoretical concern that *Candida* vaccines could disrupt the normal microbiota. Targeting antigens specific to the invasive hyphal form could minimize this potential drawback. On the host side, the diverse infection sites and the different kinds of immune deficiencies in at-risk groups are obstacles for designing a vaccine that broadly protects this wide clinical spectrum of disease^9^.

Two recombinant *Candida* vaccines have reached human clinical testing with promising results. The first, named PEV7, consists of recombinant aspartyl-proteinase 2 (Sap2), a secreted protein of *C. albicans*, assembled into virosomes^38,42^. After protection was demonstrated in *C. albicans*-challenged rats, a phase 1 clinical trial was conducted to assess the safety and immunogenicity of PEV7 in healthy female volunteers. All of the 48 vaccinated women developed specific B-cell memory responses (ClinicalTrials.gov identifier: NCT01067131)^38^.

The second vaccine, NDV-3, contains the recombinant N-terminus of *C. albicans* agglutinin-like sequence 3 protein (Als3p, a cell surface adhesin and invasin) formulated with aluminum hydroxide adjuvant^39,43^. Preclinical studies demonstrated the vaccine was immunogenic and protected mice from *Candida* species. Interestingly, mice were also protected following challenge with *Staphylococcus aureus*, apparently due to structural homology between Als3p and surface proteins on *S. aureus*. In a phase 1 clinical trial which recruited 40 volunteers, NDV-3 elicited increased antigen-specific IgG and IgA1 titers as well as increased IFN-γ and IL-17A cytokine production compared to placebo recipients (ClinicalTrials.gov identifier: NCT01273922)^39^. Based on these data and a favorable safety profile, a multicenter double-blind placebo-controlled phase 1b/2a trial was undertaken to assess the immunogenicity and efficacy of the NDV-3A vaccine in 188 women with recurrent vulvovaginal candidiasis (RVVC)^43^. NDV-3A is identical to NDV-3 except it lacks a 6-His tag and linker sequences. As with NDV-3, NDV-3A was safe and highly immunogenic. There were no statistically significant differences between treatment and placebo groups in the primary efficacy analysis. However, in a post-hoc subgroup analysis, subjects aged <40 years had significantly fewer RVVC episodes during the 12-month study period (ClinicalTrials.gov identifier: NCT01926028)^43^.

Women with RVVC represent a logical population for *Candida* vaccine studies because the incidence of recurrence is high and therefore vaccine efficacy can be determined without having to enroll inordinately large numbers of subjects in clinical trials. But it should be appreciated that in many women, RVVC is thought to be due to an overly exuberant inflammatory response to *Candida* and therefore therapeutic vaccines have the potential to worsen disease^44^. Studies to determine whether vaccines can prevent invasive candidiasis would likely need to enroll thousands of patients given the relatively low incidence even in the higher risk populations.

PEV7 and NDV-3A vaccines each were formulated with a single protein antigen. Given intraspecies and interspecies antigenic variation, including differences between yeast and hyphal forms, many investigators aim to develop multivalent *Candida* vaccines that would provide enhanced protection. This tactic has the potential to expand the protective range of the immune response, while decreasing the ability of *Candida* to evade host immunity^45^. Approaches include combining known immunogenic antigens and using in silico analysis to identify immunodominant *Candida* antigens^45,46^.

### *Cryptococcus* sp.

The species complexes of *Cryptococcus neoformans* and *C. gattii* cause cryptococcosis. Infection is thought to most commonly start after pulmonary inhalation; subsequent dissemination to other organ systems, particularly the central nervous system, can ensue. Persons with compromised T cell immunity are particularly susceptible. Annually, more than 220,000 cryptococcal meningitis cases in HIV-infected persons are estimated to occur worldwide, with about 180,000 deaths^47^. *Cryptococcus* is unique among the medically important fungi in being encapsulated; this presents opportunities and challenges in terms of vaccine development. Its polysaccharide capsule, which consists mainly of GXM, is poorly immunogenic. To improve the antigenicity of GXM, a vaccine containing GXM conjugated to tetanus toxoid was developed; immunized mice developed antibodies against GXM and were partially protected following challenge with *C. neoformans*^48^. Similarly, a conjugated peptide mimetic of GXM elicited protective antibodies^49^.

Other groups have focused on discovering protein antigens that stimulate T cell responses for inclusion in subunit vaccines. Vaccines containing crude fractions isolated from acapsular and encapsulated cryptococcal strains by a variety of techniques, including concanavalin A binding (to enrich for mannoproteins)^50^, molecular sizing^51^, alkaline extraction^52^, cellular fractionation^53^, and purification of extracellular vesicles^54^, provided some degree of protection against challenge with *C. neoformans* and *C. gattii*. More recent work has sought to discover individual antigenic proteins which protect in mouse models of cryptococcosis. Using biased and unbiased selection, our laboratory has identified 11 proteins which, when recombinantly expressed and delivered in glucan particles, protect BALB/c and/or C57BL/6 mice against an otherwise lethal infection^32,55^.

Several live-attenuated and heat-killed cryptococcal mutant strains have been proposed as vaccine candidates based on encouraging protection data. Pulmonary administration of an avirulent chitosan-deficient strain, constructed by deleting three chitin deacetylase genes, conferred full protection against a subsequent *C. neoformans* lethal infection. The vaccine elicited a protective Th1-type adaptive immune response and was effective even when heat-killed^56^. Mice were also protected from otherwise lethal challenge with mutant *C. neoformans* vaccine strains: 1) overexpressing the transcription factor, Znf2^57^; 2) lacking sterylglucosidase, which results in accumulation of the glycolipid, sterylglucoside, in the cell membrane^58^; and 3) lacking the F-box protein Fbp1^59^.

Two whole organism *Cryptococcus* vaccine approaches that provide proofs of principle, albeit with limited direct translation relevance, are worth noting. In the first, a *C. neoformans* strain was genetically engineered to express murine IFN-γ. Mice vaccinated with this strain were fully protected from subsequent challenge with a highly virulent strain by mechanisms dependent on STAT1 signaling and induction of trained immunity of dendritic cells^60,61^. In the second approach, bone marrow-derived dendritic cells were pulsed with heat-killed acapsular *C. gattii* cells and then injected intravenously into mice. The vaccine induced lung-resident memory Th17 cells and conferred protection upon recipient mice challenged with a virulent *C. gattii* strain^25^.

Finally, although closely related, the species complexes of *C. neoformans* and *C. gattii* cause infections with distinct clinical manifestations. The species elicit divergent immune responses^62^ and differ in terms of the proteome expressed during infection. Ideally, candidate vaccines able to protect against both species should be prioritized for advancement to human testing. However, for areas such as British Columbia, Canada, where hypervirulent strains of *C. gattii* are endemic, it may be practical to develop a species-specific vaccine to protect the population.

### *Aspergillus* sp.

*Aspergillus* is a globally ubiquitous filamentous fungus, widely present in soil and decaying vegetation. Airborne spores (conidia) are regularly inhaled and are typically contained by host defenses without being harmful. However, in at risk immunosuppressed individuals, germination of conidia into tissue invasive hyphae can cause a range of acute to chronic diseases. Persons most at risk include those with neutropenia, recipients of stem cell and solid organ transplants, and those receiving immunosuppressive therapy such as corticosteroids^11,63^. Essentially, these conditions pose a challenge to develop a vaccine for this target population given that the protective immunity may be diminished, together with the fact that the host needs to recognize and fight against different structures of the fungi, such as, conidia, germ tubes, and hyphae. *A. fumigatus* is the most prevalent species of *Aspergillus* responsible for opportunistic human infections, although other species, most commonly *A. flavus*, *A. niger, A. terreus*, and *A. nidulans*, can also cause disease^63^. Invasive aspergillosis is responsible for over 200,000 cases annually and associated with a high mortality rate. Furthermore, allergic manifestations can occur with sensitization to *Aspergillus* allergens, generally in patients with cystic fibrosis, severe asthma with fungal sensitization and allergic bronchopulmonary aspergillosis, the latter alone is thought to affect around 5 million people^3,63^. Therapeutic vaccines to mitigate the allergic response and bias immunity towards protective responses have promise for these difficult to treat diseases, but are still in the early development stage. Another potential *Aspergillus* vaccine market targets avian aspergillosis; outbreaks in commercial poultry flocks, particularly turkeys, can have major economic consequences^64^.

Vaccination studies in mice have demonstrated protection using crude and recombinant *Aspergillus* antigens delivered using a variety of routes and adjuvants (reviewed in the ref. ^65^). When examined, protection generally required CD4^+^ T cells. Dendritic cells pulsed with conidia or conidial RNA also conferred Th1-mediated antifungal resistance in a mouse model of allogeneic hematopoietic transplantation^66^. These studies informed innovative studies in humans undergoing allogenic hematopoietic transplant, where donor T cells can be expanded ex vivo with *Aspergillus* antigens and adoptively transferred into the patient. *Aspergillus*-specific CD4^+^ and IFN-γ-producing T cell clones, generated by incubating peripheral blood mononuclear cells with heat-killed conidia, were adoptively transferred to transplant recipients with evidence of invasive aspergillosis. Encouragingly, 9 of 10 patients who received adoptive T cells resolved their infection, compared to 7 of 13 control patients who did not receive the immunotherapy^67^. A more efficient method for ex vivo expansion of donor T-cell populations using recombinant *A. fumigatus* proteins and selection based on expression of the activation markers CD154 and CD137 has been described^68^. Cross-reactivity to other species was observed suggesting this could be part of a broad approach towards therapeutic vaccination in immunocompromised patients with mold infections^68^. Another novel approach worthy of mention, although not technically a therapeutic vaccine, is the use of chimeric antigen receptor (CAR) T cells genetically modified for fungal specificity. CAR T cells expressing the β-1,3-glucan receptor Dectin-1 bound to and inhibited *A. fumigatus*^69^.

The above studies looked at vaccines that protected largely by eliciting T cell-mediated immunity. Surprisingly, few studies have focused on vaccines designed to elicit protective antibodies. That this approach has merit is suggested by studies showing extended survival of mice that received passive administration of monoclonal antibodies directed to cell surface antigens of *A. fumigatus*^65,70^. In accordance, exploiting an observation that some sialylated oligosaccharide structures are present in both group B *Streptococcus* (GBS) and *A. fumigatus*, passive transfer of a GBS-specific monoclonal antibodies, and vaccination with GBS improved survival in mouse models of invasive aspergillosis^71^.

### Endemic mycoses

Endemic mycoses are caused by dimorphic fungi of the Ascomycota phylum, including species of *Histoplasma*, *Coccidioides*, *Blastomyces*, *Paracoccidioides*, *Talaromyces*, and *Emergomyces*. After infectious spores encounter mammalian hosts, temperature-induced phase transition to tissue-invasive yeasts, or for *Coccidioides*, spherules, occurs^72^. Three factors make the endemic mycoses attractive candidates for vaccine development. First, they cause significant morbidity in apparently immunocompetent individuals. Second, a vaccine could be targeted to those at risk due to residence in or travel to the geographic areas where the mycosis is found. Third, the causative fungi are genetically related which increases the feasibility of developing cross-protective vaccines.

A formalin-killed spherule (FKS) vaccine for coccidioidomycosis was studied in a human clinical trial after it showed promising results in mice and Rhesus monkeys. A Phase 3 study randomized 2867 healthy volunteers from California and Arizona; groups received three intramuscular injections of either FKS vaccine or sterile NaCl solution. Unfortunately, no significant differences were observed between groups in terms of clinical endpoints, and the vaccinated groups had greater local and systemic adverse reactions^73^. Nevertheless, the study demonstrated the feasibility of conducting human trials of fungal vaccines. In the field of veterinary medicine, a genetically engineered live-attenuated strain of *B. dermatitidis*, lacking the major virulence factor BAD-1, was safe, well-tolerated, and immunogenic in dogs^74^. Studies to determine vaccine efficacy in canine blastomycosis are still needed, although in experimental models, the vaccine did protect mice from lethal infection.

Given their theoretical safety advantages, identification of protective antigens for use in subunit vaccines has been the focus of much research. One of the earliest candidate antigens identified was heat shock protein (Hsp) 60. Vaccination with recombinant Hsp60 induced protective immunity against *Histoplasma* and *Paracoccidioides* following pulmonary infection in mice^75,76^. Fungal and mammalian Hsp are evolutionarily conserved; this raises theoretical concerns about untoward autoimmune responses unless homologous regions are edited out. Other recombinant antigens, many of which have no significant human homologies, have protected mice in experimental models of endemic mycoses^77–79^. A multivalent vaccine containing three recombinant antigens elicited protective responses in mice challenged with *Coccidioides*^80^. Moreover, when the peptides of these proteins that elicited the best T cell responses were combined into a recombinant epitope-based vaccine, enhanced survival, reduced fungal burden, and robust Th1 and Th17 immune responses were observed^81^. Use of chimeric proteins reduces the expense of manufacturing and testing vaccines, but could generate unwanted immune responses to the created neoantigens.

### Pan-fungal vaccines

The above vaccine strategies are mainly focused on preventing specific mycoses. The “holy grail” is a pan-fungal vaccine that protects against many if not most of the broad range of systemic fungal infections seen in the human population. In this regard, several vaccine candidates are worthy of mention. As noted above, nearly all medically important fungi possess a cell wall that contains β-glucans. Immunization of mice with β-glucans conjugated to the carrier protein CRM197 resulted in protection against challenge with taxonomically distant fungal pathogens, including *C. albicans*, *A. fumigatus*, and *C. neoformans*^82,83^. Protection was correlated with an antibody response directed against β-1,3-glucan, but not β-1,6-glucan^84^. Similarly, mice given a subcutaneous inoculation containing whole heat-killed *Saccharomyces cerevisiae* yeast cells were protected against a subsequent challenge with fungi from five genera; the mechanism is uncertain but postulated to require T-cell adaptive responses^85,86^. Mice vaccinated with the aforementioned *C. neoformans* strain deleted of the F-box protein Fbp1^59^ are cross-protected against *C. gattii*, *Aspergillus fumigatus*, and *Candida albicans*, suggesting its potential to act as a pan-fungal vaccine^59^.

Interestingly, β-glucans are strong stimulators of “trained immunity”, a process whereby activation of innate immunity leads to epigenetic changes that enhance responses to subsequent infections^87^. The contribution of trained immunity to the cross-protection seen with pan-fungal vaccines which contain β-glucans merits further study.

The chaperone protein calnexin contains a 13-amino acid sequence which is highly conserved among fungi of the *Ascomycota*, a phylum that contains many medically important fungi including *Aspergillus* and the endemic dimorphic fungi. Vaccine delivery of calnexin in glucan particles conferred immunity to lethal challenge with multiple ascomycetes via expansion of antigen-specific CD4^+^ T cells^33^. These results suggest a strategy whereby T or B cell epitopes common to multiple species of fungi could be identified and combined to yield pan-fungal vaccines.

## Conclusions

Remarkable progress has been made towards the development of fungal vaccines for use in humans. In animal studies, protection against all the major medically important mycoses has been achieved using a variety of vaccine designs ranging from subunit formulations to live attenuated fungi. Superior efficacy has been demonstrated using novel adjuvants and delivery systems aimed at stimulating arms of the immune system critical for control of fungal invasion. Three vaccines have undergone human trials demonstrating the feasibility of performing clinical trials targeting at risk populations. While many scientific and logistical obstacles remain, there is reason to be optimistic that clinically approved fungal vaccines will be forthcoming.
